# Post-traumatic Brodie’s Abscess of the Tarsal Cuboid: A Case Report and Review of the Literature

**DOI:** 10.7759/cureus.53158

**Published:** 2024-01-29

**Authors:** Ioannis K Tzellios, Dimitrios I Gelalis, Ioannis Gkiatas, Emilios Ε Pakos, Ioannis D Gelalis

**Affiliations:** 1 Department of Orthopaedic Surgery and Traumatology, University Hospital of Ioannina, Ioannina, GRC

**Keywords:** brodie's abscess, escherichia coli, curettage, penetrating trauma, infection, tarsal cuboid, chronic sub-acute osteomyelitis

## Abstract

Brodie's abscess of the tarsal cuboid is a relatively rare presentation of this disease. In this study, we present the case of a 20-year-old male with post-traumatic Brodie’s abscess of the tarsal cuboid that was left untreated for three years after the traumatic episode (penetrating injury with a sharp piece of wood). The patient presented pain over the injured area, limping, while plain foot radiographs showed a small lytic cavitary area in the cuboid. The magnetic resonance imaging revealed the presence of the abscess in a 2-cm diameter cavity in the cuboid bone and chronic inflammation of the surrounding plantar musculature. The treatment regime included curettage of the cavity, debridement of the inflammatory tissues, and administration of antibiotics, according to the cultures harvested intraoperatively, for six weeks. During this period, symptoms completely resolved.

## Introduction

Brodie’s abscess is a rare form of chronic pyogenic sub-acute osteomyelitis of the bones, with an insidious onset usually and without obvious clinical symptomatology, which was first described by Sir Benjamin Brodie in 1832 [1-10]. Most of the cases involve the metaphysis, epiphysis, or diaphysis of the long bones of the lower limb such as the femur or the tibia [1,2,4,7]. However, Brodie’s abscess can be detected in other anatomical areas as well, including small bones such as those of the tarsus. Its characteristic radiographic appearance consists of a well-defined radiolucent cavity with a sclerotic margin [8] while magnetic resonance imaging (MRI) reveals the abscess cavity (‘penumbra sign’) [9]. Cases of Brodie’s abscess of the tarsal cuboid are rarely described in the medical literature [2].

In the present case report, an uncommon case of a post-traumatic Brodie’s abscess of the tarsal cuboid is presented, caused by a penetrating injury, in a 20-year-old male patient with a 12-month follow-up, and a review of the available literature is provided. This case report has been reported in line with the Surgical Case Report (SCARE) criteria.

## Case presentation

A 20-year-old male presented at the Orthopedic outpatient department of our hospital with the primary complaint of pain associated with difficulty weight-bearing on his right foot. He had no history of chronic conditions, but he is a smoker (20 cigarettes/day). He had a working accident three years ago at a construction site when he suffered from a penetrating injury from a sharp object although he was wearing protective footwear. No fracture was identified on the radiographs. He was admitted to the local hospital where he was treated surgically. His wound was debrided, cleaned, and sutured, and he was administered broad-spectrum antibiotics intravenously. The patient was discharged with oral antibiotic treatment.

For three years postoperatively, the patient experienced persistent pain at the trauma site and was treated occasionally with oral nonsteroid anti-inflammatory drugs (NSAIDs), local steroid injections, and rest. The patient visited our outpatient department with inflammation around the injured area, localized pain on the lateral aspect of the midfoot, and inability to bear his total body weight on his right foot. The visual analog scale (VAS) score at rest was 5/10 and increased up to 9/10 after a few minutes of walking.

The patient underwent plain radiographs of his right foot in anteroposterior (AP) and oblique views, a computed tomography (CT) scan, and magnetic resonance imaging (MRI) with contrast. Before his admission blood tests showed normal inflammation markers (C-reactive protein (CRP) and erythrocyte sedimentation rate (ESR)), slightly elevated alanine transaminase (ALT), and aspartate aminotransferase (AST) levels due to chronic consumption of NSAIDs. His severe acute respiratory syndrome coronavirus 2 (SARS-CoV-2) test was negative. The chest radiograph was normal.

**Figure 1:**
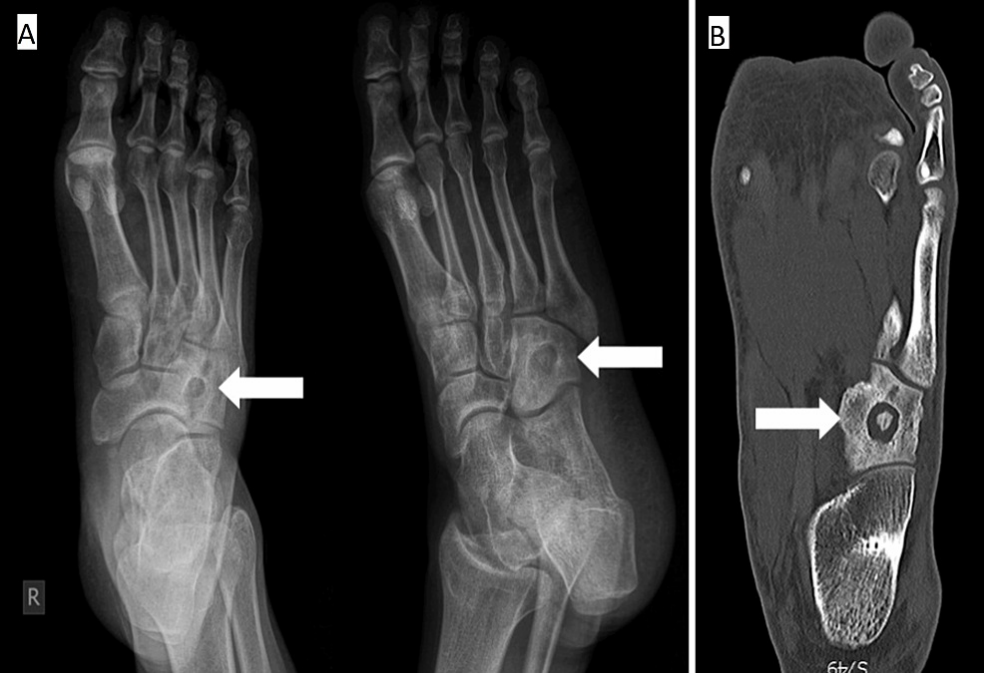
(A) Anteroposterior (AP) and oblique view radiographs; (B) CT scan of the right foot A well-defined osteolytic cavitary lesion in the tarsal cuboid is revealed (arrows).

Right foot radiographs and the CT scan revealed an osteolytic cavitary lesion in the central part of the tarsal cuboid bone with a sclerotic rim and no new bone formation in the cavity (Figure 1).

**Figure 2:**
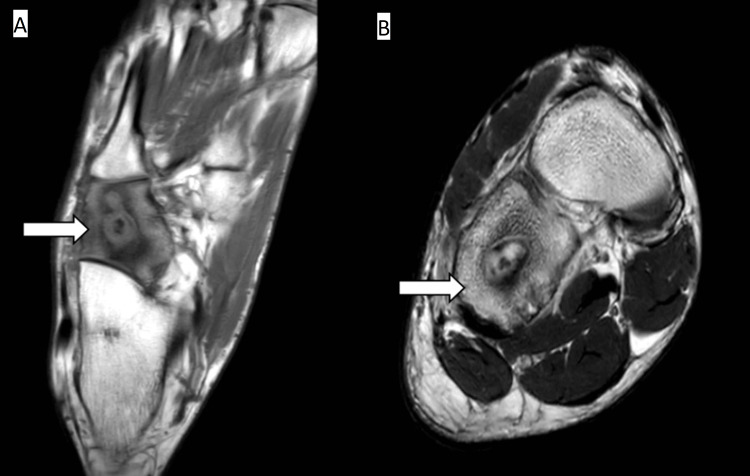
(A) Transverse T1-weighted MRI image; (B) coronal T2-weighted MRI image Cavitary lesion in the tarsal cuboid with high signal intensity margins and low signal intensity in the center (arrows)

Consequently, the patient underwent an MRI with contrast, which revealed a pathological signal of the cuboid in all sequences and edema of the surrounding soft tissues. Moreover, a cavity with a 2 cm diameter was observed in the center of the cuboid with higher signal intensity margins than in the center, implying the well-known penumbra sign (Figure 2).

**Figure 3:**
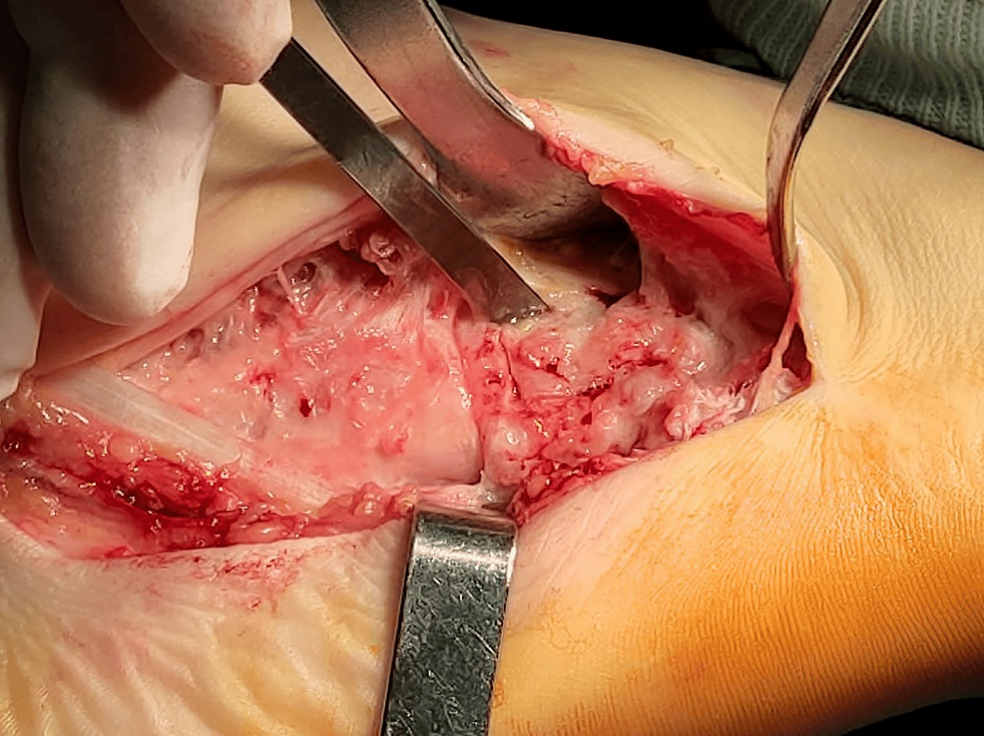
Bone window opening to the superior aspect of the tarsal cuboid

**Figure 4:**
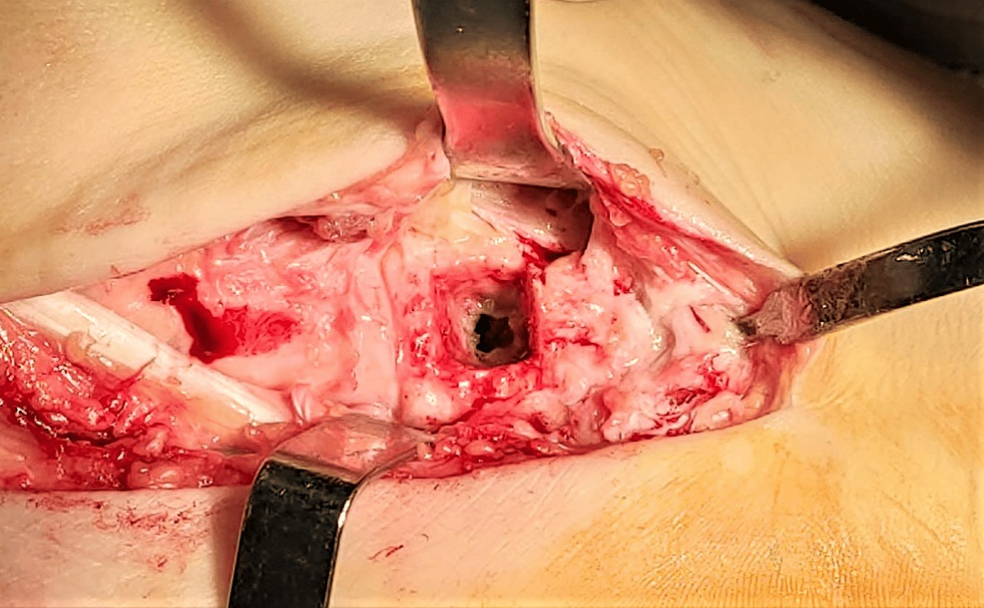
Cavity opened, excised, and lavaged

**Figure 5:**
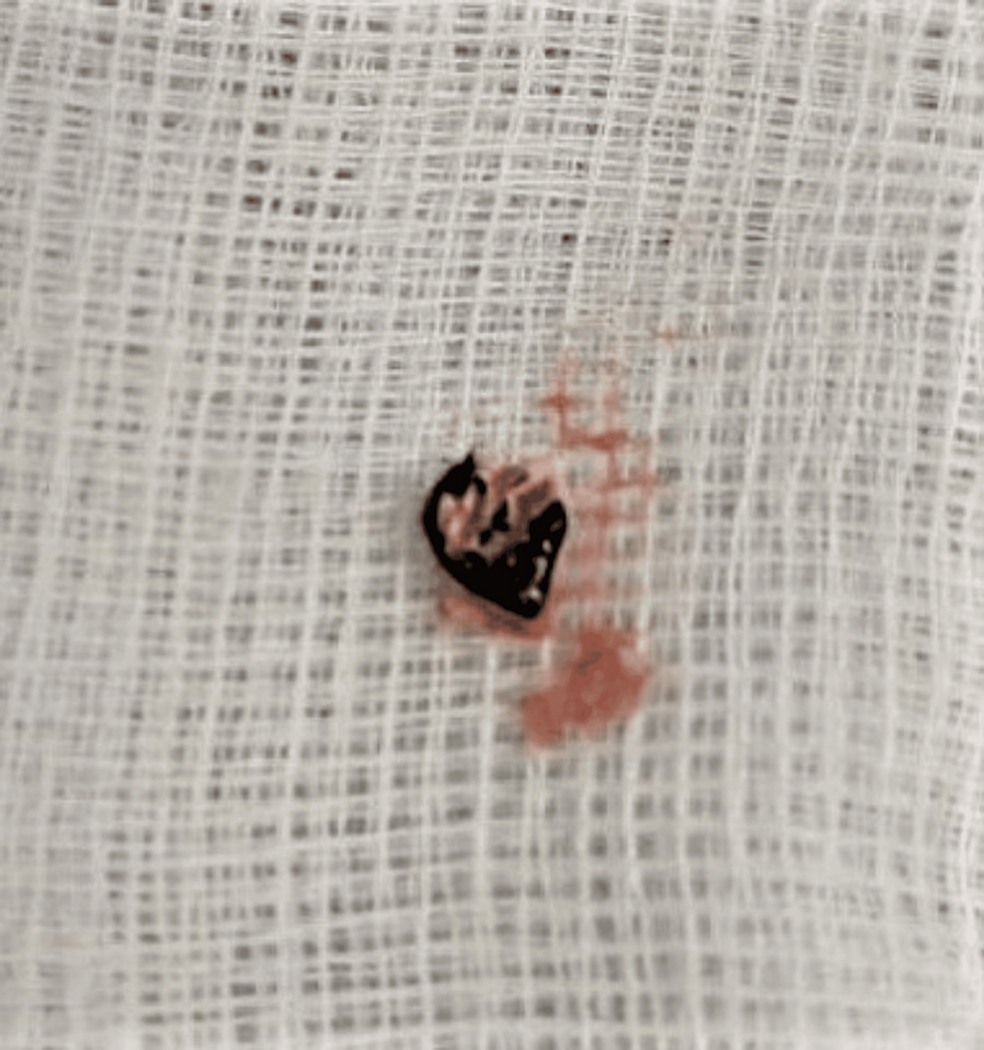
Foreign body (probably shoe sole remnant) removed from the tarsal cuboid bone cavity

After consideration of all clinical and radiological data, surgical treatment was proposed. The patient was taken to the operating room, where he was positioned in a supine position, under general anesthesia. An inflatable tourniquet on his right thigh was used for exsanguination. The tarsal cuboid was exposed through a dorsolateral approach of the right foot with an incision of approximately 5 cm. Α bone window 1.5x1.5 cm was created with a chisel over the cuboid (Figures 3, 4), followed by irrigation of the pus inside the cavity, where a foreign body of elastic consistency (probably shoe sole remnant) was identified and removed (Figure 5). Finally, the cavity wall was meticulously excised with a curette and repeatedly lavaged with normal saline and diluted povidone solution. Pus and part of the excised bone were sent for aerobic and anaerobic cultures. The wound was closed with surgical staples and a Robert-Jones dressing was applied.

**Figure 6:**
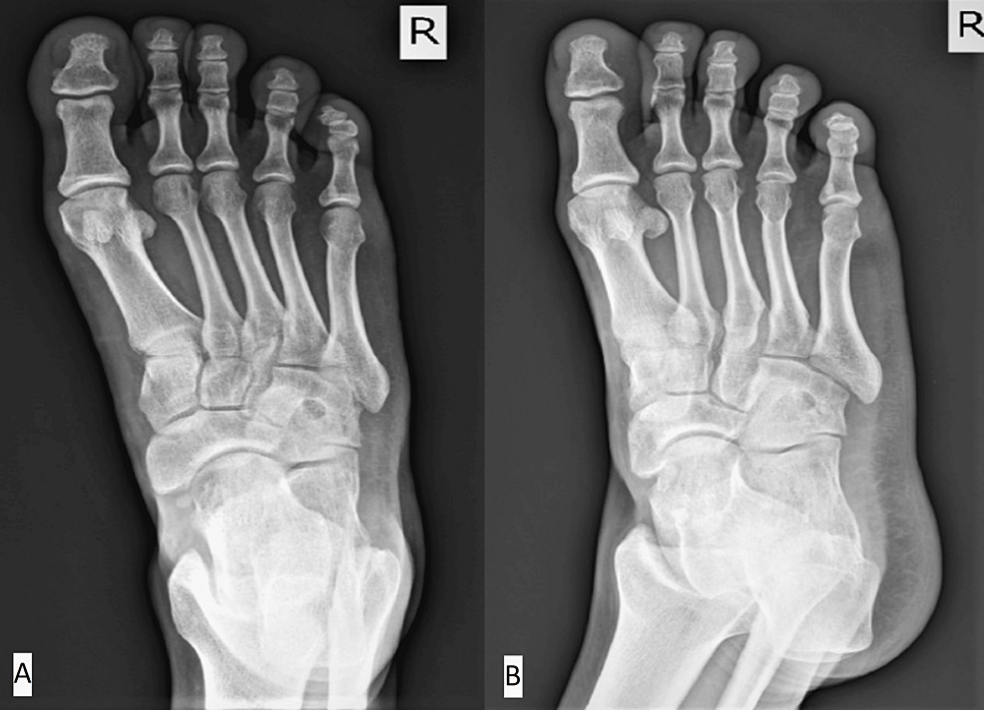
(A) Anteroposterior (AP) view radiograph; (B) oblique view radiographs Partial new bone formation in the cavity of the tarsal cuboid of the right foot.

Postoperatively, no weight bearing of the right foot was allowed. Cultures were positive after 72 hours for Escherichia coli resistant to ciprofloxacin. According to the antibiogram, the patient was administered intravenous Cefoxitin and Amikacin for two weeks and amoxicillin and clavulanic acid per os. for another four weeks. Sutures were removed on day 15 postoperatively without wound complications. The patient was advised for partial weight bearing of the right foot for six weeks postoperatively. He was free of symptoms and walked without any difficulty after that period. A radiological exam revealed partial new bone formation within the previous cavity of the tarsal cuboid 12 months postoperatively (Figure 6).

## Discussion

Brodie’s abscess is described as subacute osteomyelitis that can affect all ages but more often males at a young age. Patients usually present localized pain that progressively worsens. Reports in the literature mention an average of 12 weeks of presenting symptoms before a concrete diagnosis is formed. According to Niels van der Naald et al., serum inflammation markers were within the normal range or mildly elevated, which is compatible with our case report [1]. For the diagnosis of Brodie’s abscess, the imaging choices are plain radiographs, CT scans, and MRI. MRI is the gold standard for the final diagnosis of Brodie’s abscess at younger ages. Based on the findings of Niels van der Naald et al.'s study, the most common pathogen found in Brodie’s abscess is Staphylococcus Aureus [1]. Comparing our case with the aforementioned review, the pathogen of Escherichia coli, which our patient’s cultures showed, is one of the rarest as far as it concerns Brodie’s abscess.

Furthermore, it has been reported that surgery is the treatment of choice for Brodie’s abscess. According to Özbek EA et al., the combination of debridement, irrigation, and antibiotic-loaded cement spacers was the best treatment for their patient, with the result of full union [4]. In all the described case reports in the literature, the proposed treatment was debridement and irrigation of the cavity of the abscess. However, the consideration of using antibiotic-loaded cement spacers lies with the surgeon.

According to the classification of Patzakis et al., which divides the foot into three zones, the least probable zone for bone or joint damage from a penetrating injury was zone 2 because of the metatarsal arch and the abundant soft tissue that provides protection [11]. Zone 1 and Zone 3 were the most prone to a penetrating injury because of the weight-bearing factor. The fact that, in our case, the injury occurred in Zone 2 makes our case report even rarer.

## Conclusions

Brodie’s abscess is a rare form of chronic pyogenic sub-acute osteomyelitis of the bone. The presence of Brodie’s abscess in the cuboid tarsal bone is rare. Symptoms include localized pain and weight-bearing difficulty on the affected foot. Appropriate imaging and blood examinations contribute to the right diagnosis. Surgical treatment of Brodie's abscess, including debridement and irrigation in combination with the appropriate antibiotics, consists of the treatment option with the most promising outcomes.
